# IUC26645-88 Challenges of AI in Surgical Planning for Radical Prostatectomy: Barriers and Progress

**DOI:** 10.1093/oncolo/oyag286.011

**Published:** 2026-08-21

**Authors:** F Rajaee Rizi, M S Jamadi

**Affiliations:** Endocrine and Metabolism Research Center, Isfahan University of Medical Sciences, Isfahan, Iran - Iran, Islamic Republic of; Department of Obstetrics and Gynecology, Faculty of Medicine, Isfahan University of Medical Sciences, Isfahan, Iran - Iran, Islamic Republic of

## Abstract

**Background:**

Artificial intelligence (AI), encompassing machine learning (ML) and deep learning (DL), holds transformative potential across the continuum of radical prostatectomy (RP) surgical planning. This narrative review comprehensively examines progress made to date and characterises the barriers currently impeding clinical translation.

**Methods:**

A systematic literature search was conducted across PubMed/MEDLINE, Scopus, Web of Science, and Scholar Gateway, covering January 2015 to June 2026. Evidence was synthesised narratively across five domains: preoperative imaging and anatomical reconstruction; preoperative risk stratification and nerve-sparing decision-making; intraoperative guidance and real-time margin assessment; postoperative outcome prediction; and barriers to clinical implementation. Reporting followed SANRA criteria.

**Results:**

In preoperative imaging, DL-based prostate segmentation achieved Dice similarity coefficients >0.91 on benchmark datasets, and integrated radiomics-clinical models for extraprostatic extension (EPE) prediction achieved pooled AUC 0.89 (95% CI: 0.86–0.92), sensitivity 0.83 (95% CI: 0.78–0.87), and specificity 0.82 (95% CI: 0.77–0.86) in meta-analytic synthesis. Explainable ML for side-specific EPE achieved AUROC 0.81, outperforming existing nomograms (AUROC 0.75–0.76). Intraoperatively, a DL model for automated fluorescence confocal microscopy (FCM) image classification achieved AUC 0.93 (internal) and 0.83 (external validation), providing a scalable alternative to NeuroSAFE in under 2 seconds without on-site pathology expertise. DL applied to intraoperative video predicted urinary continence recovery at 3 months with AUC 0.882, sensitivity 92.2%, and accuracy 85.3%. Postoperatively, supervised ML outperformed validated nomograms for biochemical recurrence (BCR) prediction at 1, 3, and 5 years (AUC 0.894, 0.876, 0.894 vs. 0.815, 0.798, 0.799 for best nomogram). A multimodal AI digital pathology system achieved NCCN guideline inclusion as an adjunct risk stratification tool. Despite these advances, critical barriers persist: small heterogeneous single-centre training datasets, lack of external validation, “black box” model architectures incompatible with surgical transparency requirements, algorithmic bias from non-representative populations, absent unified regulatory frameworks, data privacy constraints, and insufficient prospective clinical evidence of patient-level benefit.

**Conclusions:**

AI demonstrates robust proof-of-concept across the full RP surgical planning continuum. Transition to routine deployment requires prospective multicentre workflow-embedded trials, explainable architectures, demographically diverse training data, federated learning approaches, and cross-disciplinary regulatory collaboration.

